# Supplementary material for: Heliotropium procubens Mill: Taxonomic Significance and Characterization of Phenolic Compounds via UHPLC–HRMS- In Vitro Antioxidant and Enzyme Inhibitory Activities
Source: Molecules. 2023 Jan 19;28(3):1008. doi: 10.3390/molecules28031008 (PMC9921235; doi:10.3390/molecules28031008)
Supplement: Supplementary file 1 [file molecules-28-01008-s001.zip › molecules-2138940-supplementary.pdf]

molecules

MDPI

SUPPLEMENTARY FILE

Heliotropium procubens Mill: Taxonomic significance and characterization of phenolic compounds via UHPLC-HRMS- *In vitro* antioxidant and enzyme inhibitory activities

Kalliopi-Maria Ozntamar-Pouloglou [1], Antigoni Cheilari [1], Gokhan Zengin [2], Konstantia Graikou [1], Christos Ganos [1], George-Albert Karikas [3,*], Ioanna Chinou [1,*]

[1] Lab. of Pharmacognosy & Chemistry of Natural Products, Faculty of Pharmacy, National and Kapodistrian University of Athens, Panepistimiopolis, Zografou, 15771, Greece

[2] Dept of Biology, Science Faculty, Selcuk University, Konya, Turkey

[3] Dept of Biomedical Sciences, University of West Attica, Greece

*Correspondence: ichinou@pharm.uoa.gr; karikasg@uniwa.gr

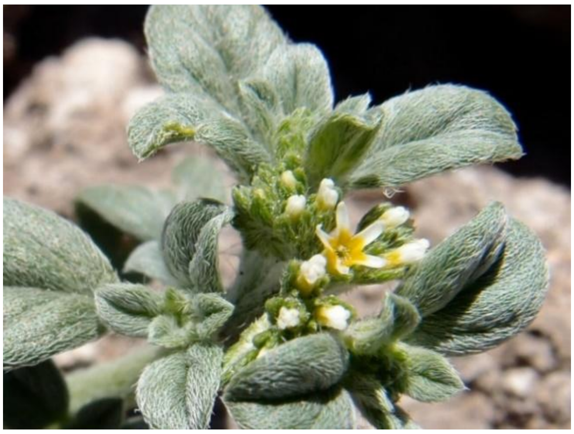

Photo 1: *Heliotropium procubens* Mill.

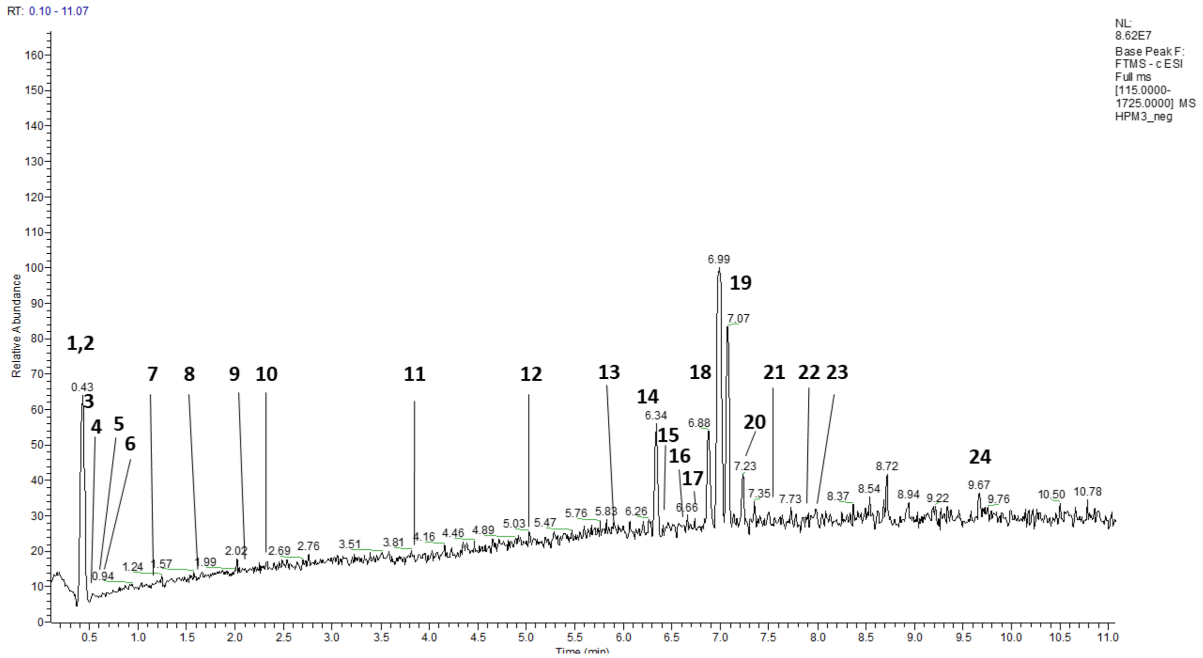

Figure S1: Characterization of the phenolic constituents of *Heliotropium procubens* extract by UHPLC-MS.

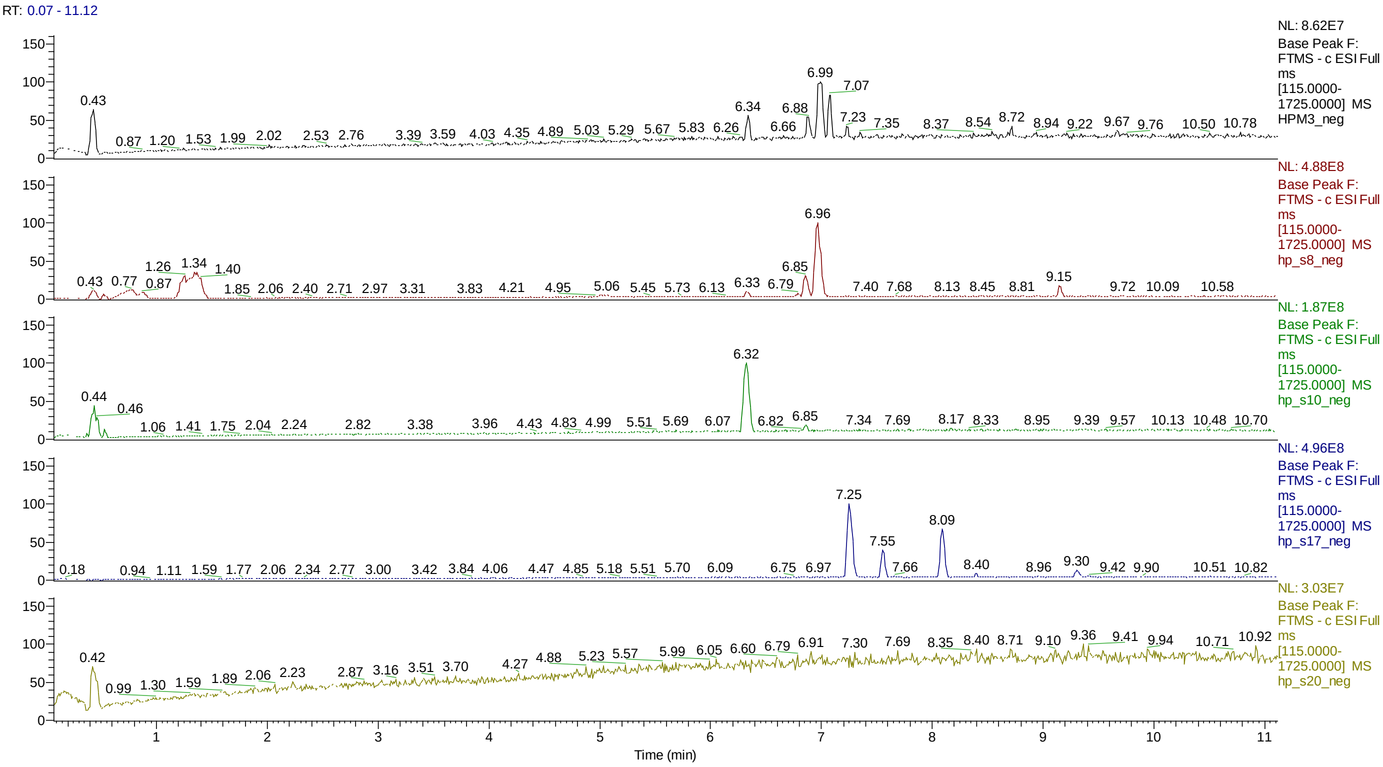

Figure S2: UHPLC-MS chromatogram of *Heliotropium procubens* extract (HPM3, top chromatogram) and fractions (HP8, HP10, HP17, HP20).

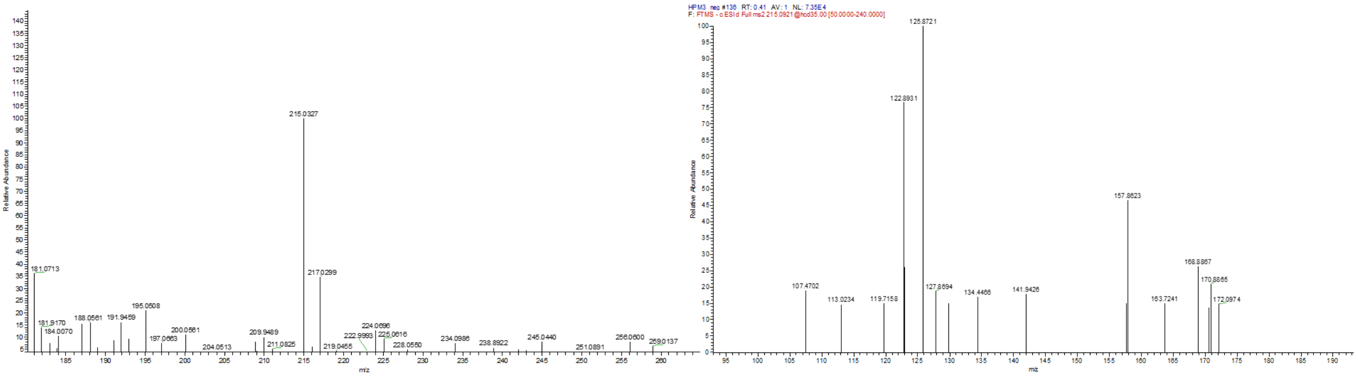

Figure S3: Spectra of **3**: Left: Mass spectrum of **3** (m/z = 215); Right: MS2 spectrum of **3** (at m/z = 215).

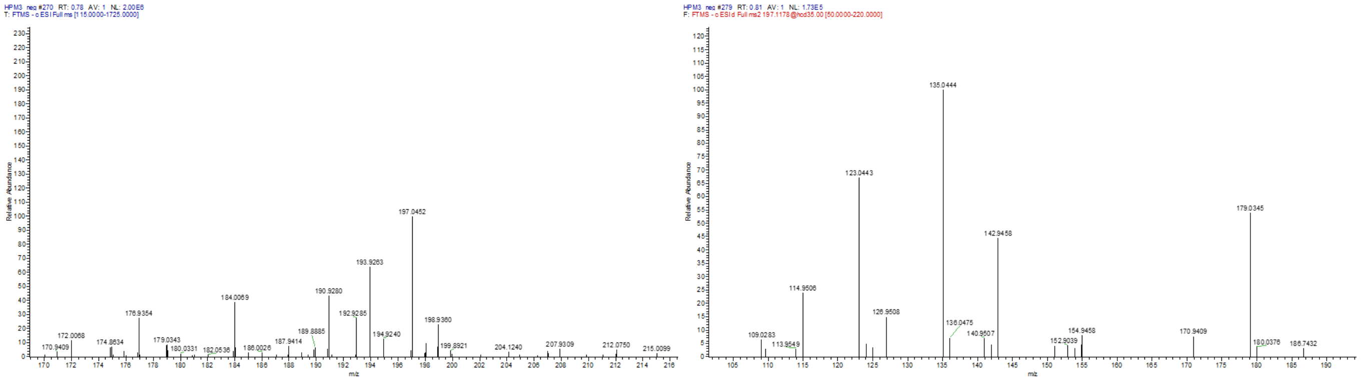

Figure S4: Spectra of **4:** Left: Mass spectrum of **4** (m/z = 197); Right: MS2 spectrum of **4** (at m/z = 197).

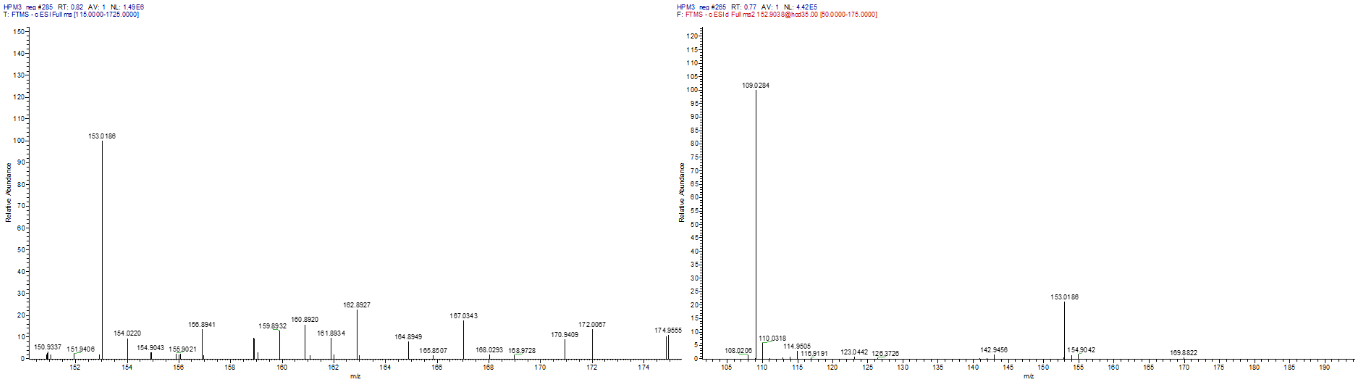

Figure S5: Spectra of **5:** Left: Mass spectrum of **5** (m/z = 153); Right: MS2 spectrum of **5** (at m/z = 153).

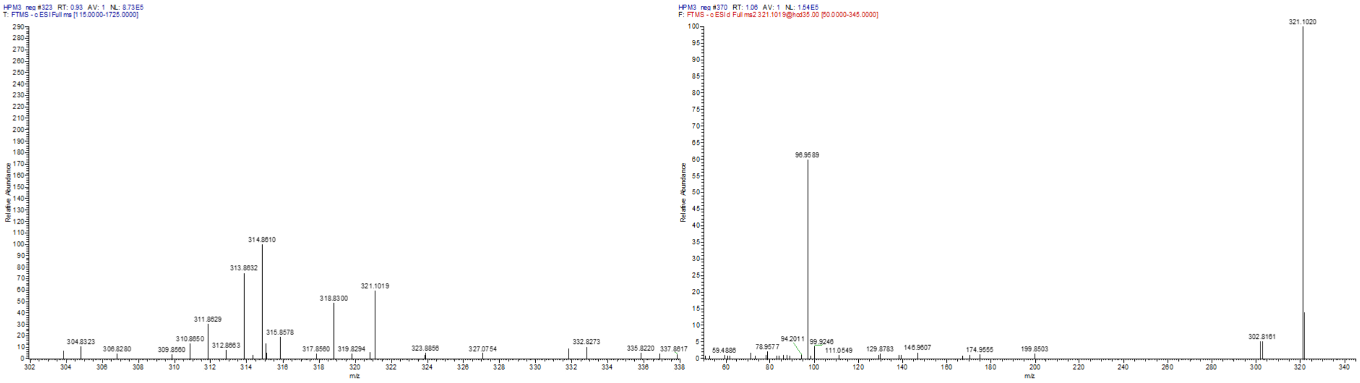

Figure S6: Spectra of **6:** Left: Mass spectrum of **6** (m/z = 321); Right: MS2 spectrum of **6** (at m/z = 321).

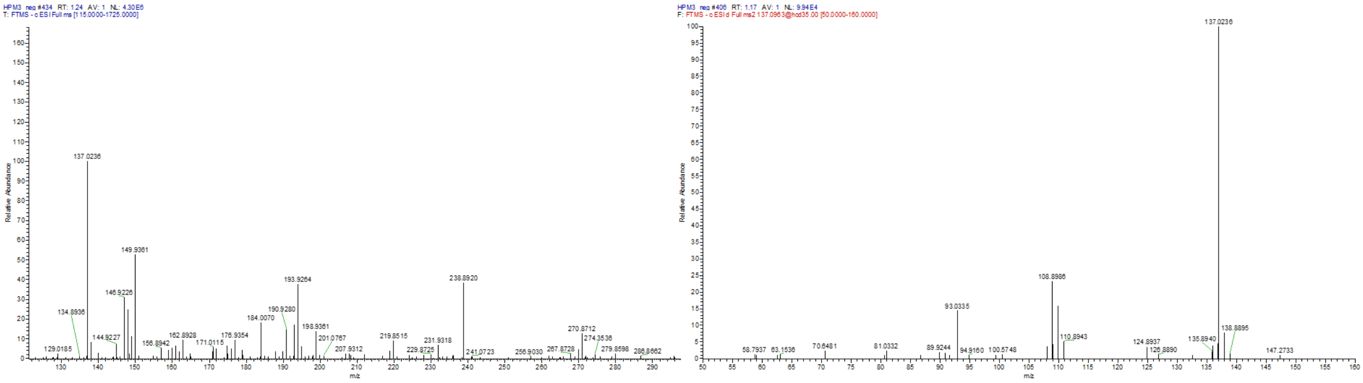

Figure S7: Spectra of **7:** Left: Mass spectrum of **7** (m/z = 137); Right: MS2 spectrum of **7** (at m/z = 137).

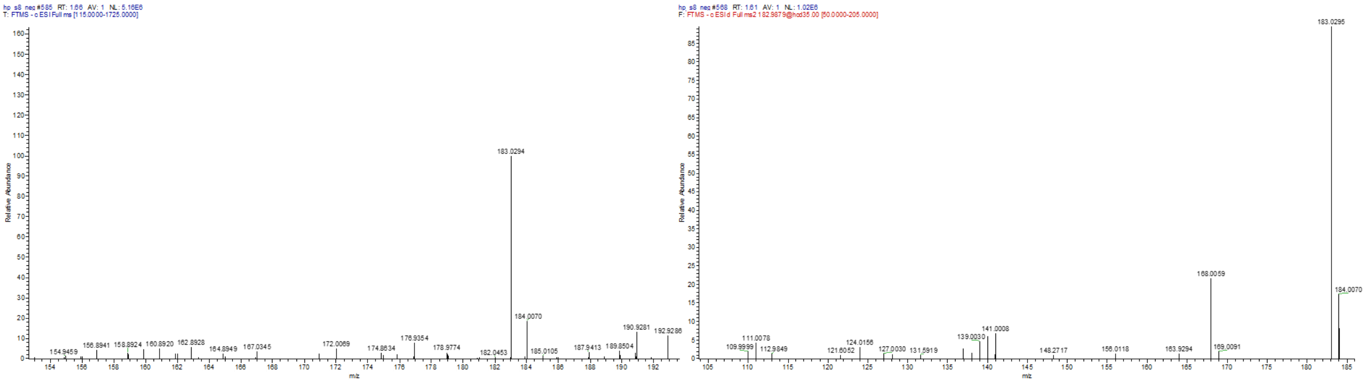

Figure S8: Spectra of **8:** Left: Mass spectrum of **8** (m/z = 183); Right: MS2 spectrum of **8** (at m/z = 183).

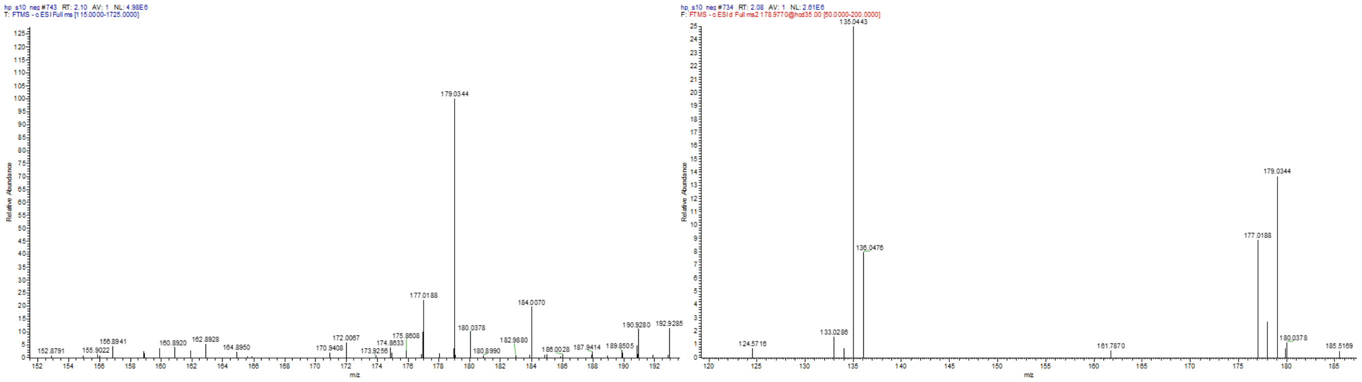

Figure S9: Spectra of **9:** Left: Mass spectrum of **9** (m/z = 179); Right: MS2 spectrum of **9** (at m/z = 179).

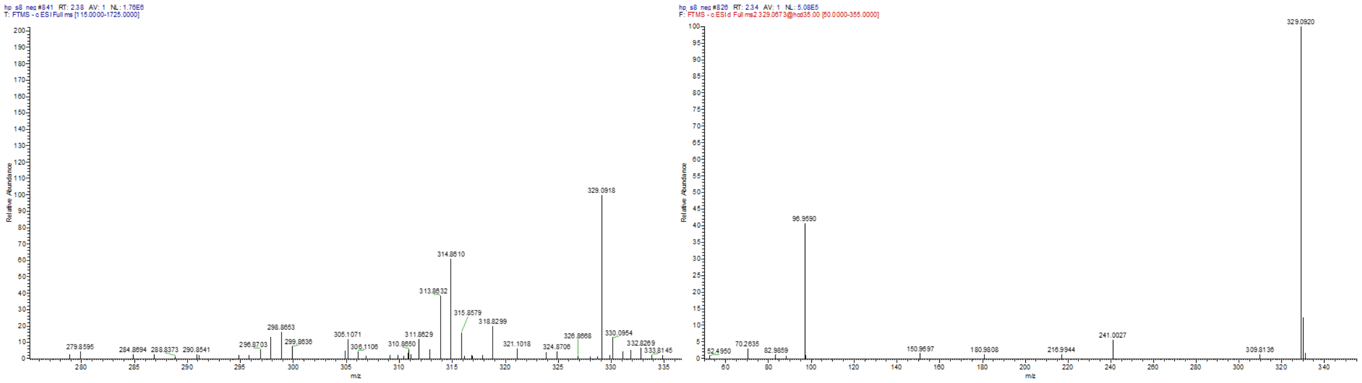

Figure S10: Spectra of **10:** Left: Mass spectrum of **10** (m/z = 329); Right: MS2 spectrum of **10** (at m/z = 329).

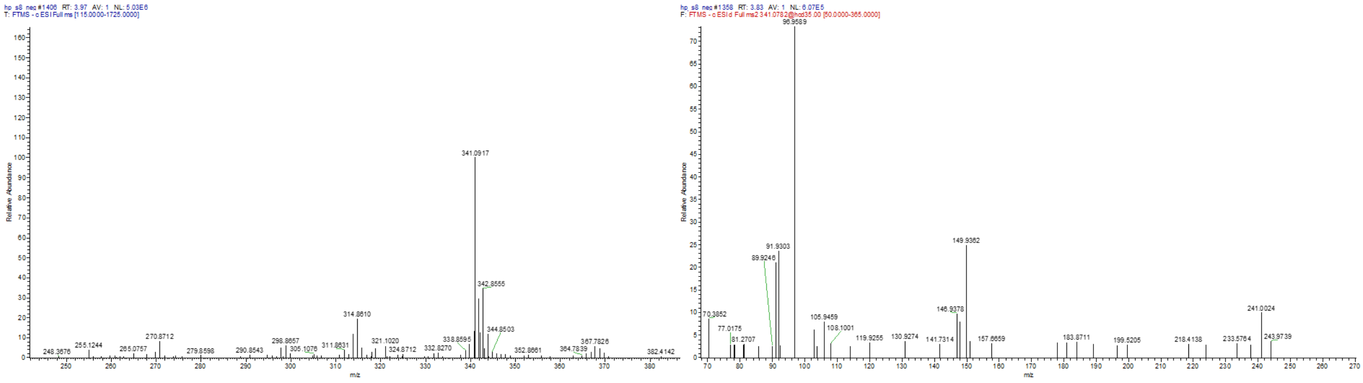

Figure S11: Spectra of **11:** Left: Mass spectrum of **11** (m/z = 305); Right: MS2 spectrum of **11** (at m/z = 305).

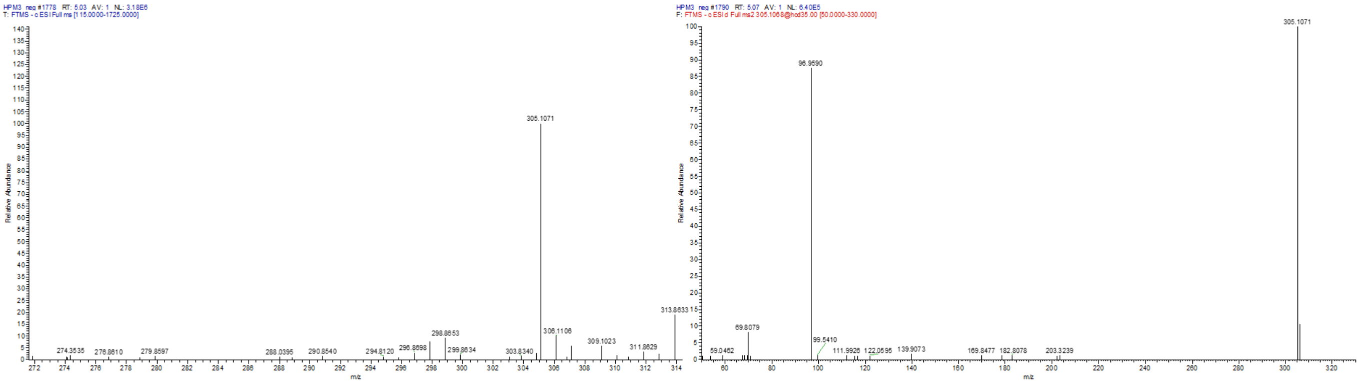

Figure S12: Spectra of **12:** Left: Mass spectrum of **12** (m/z = 341); Right: MS2 spectrum of **12** (at m/z = 341).

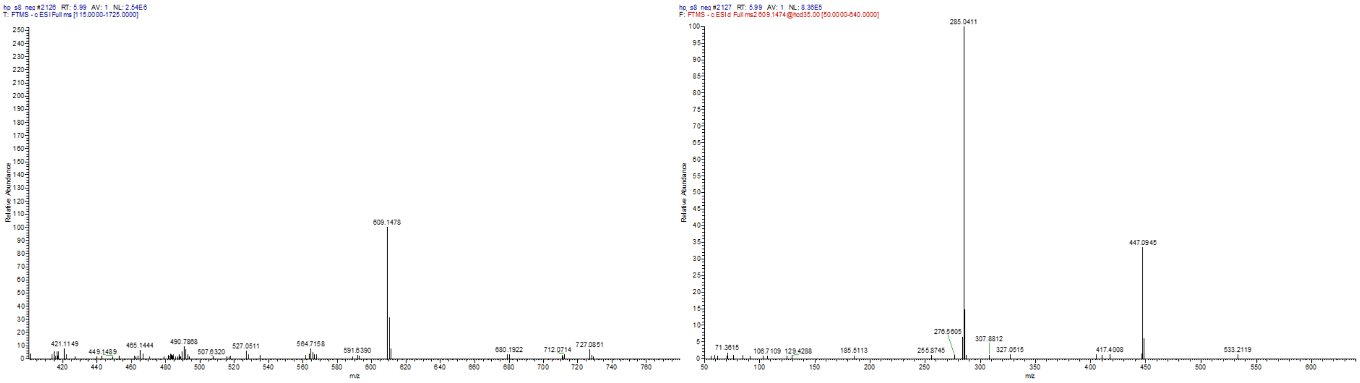

Figure S13: Spectra of **13:** Left: Mass spectrum of **13** (m/z = 609); Right: MS2 spectrum of **13** (at m/z = 609).

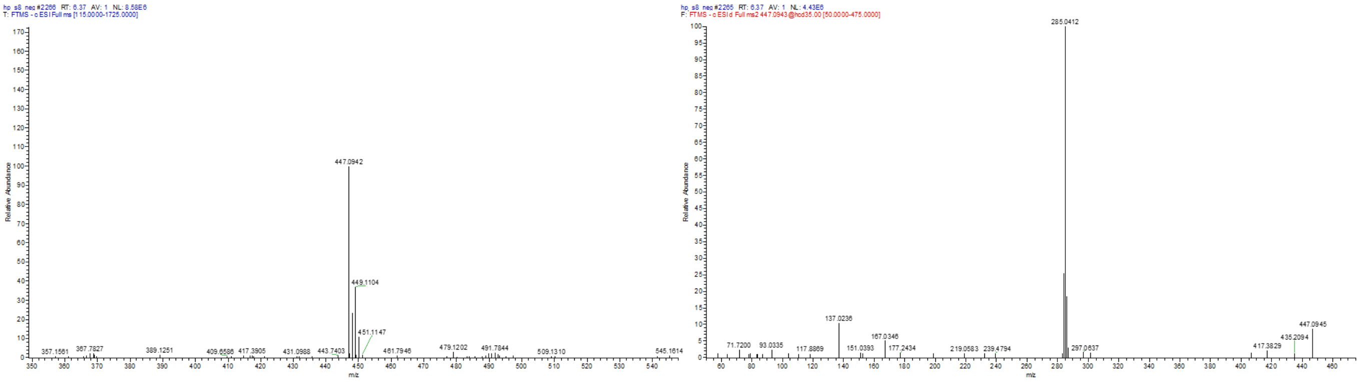

Figure S14: Spectra of **14:** Left: Mass spectrum of **14** (m/z = 447); Right: MS2 spectrum of **14** (at m/z = 447).

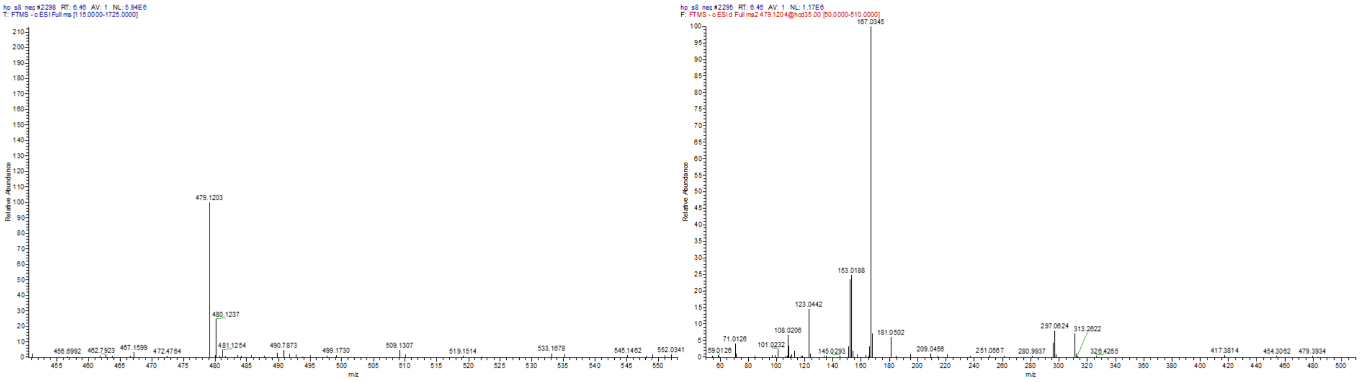

Figure S15: Spectra of **15:** Left: Mass spectrum of **15** (m/z = 479); Right: MS2 spectrum of **15** (at m/z = 479).

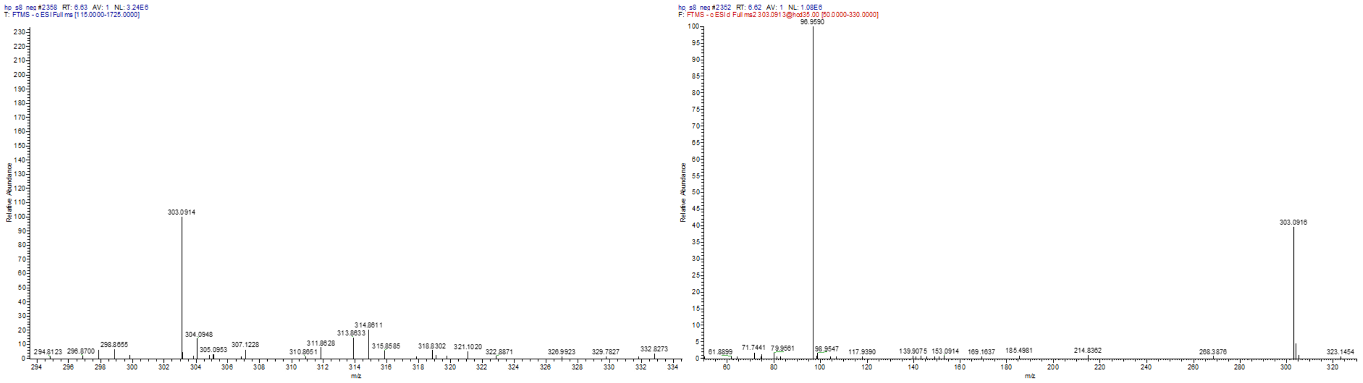

Figure S16: Spectra of **16:** Left: Mass spectrum of **16** (m/z = 303); Right: MS2 spectrum of **16** (at m/z = 303).

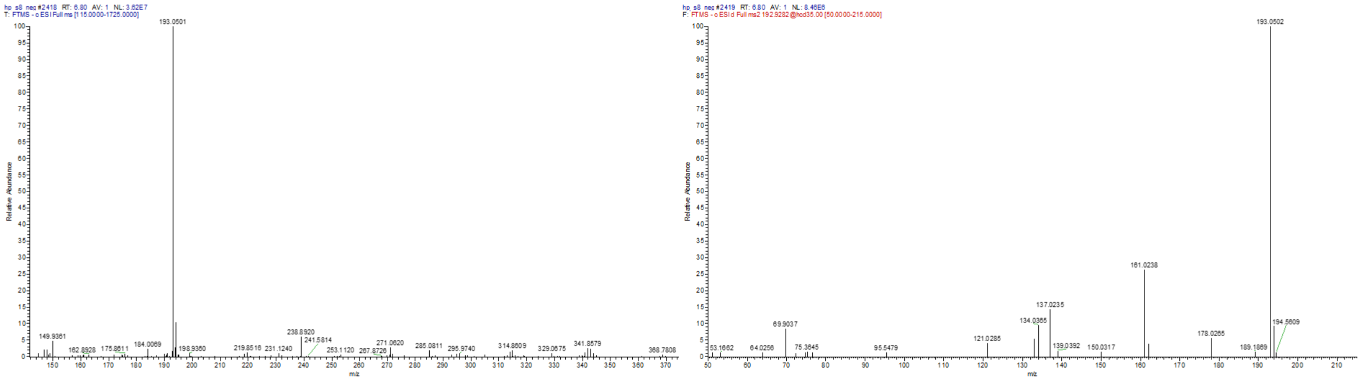

Figure S17: Spectra of **17:** Left: Mass spectrum of **17** (m/z = 193); Right: MS2 spectrum of **17** (at m/z = 193).

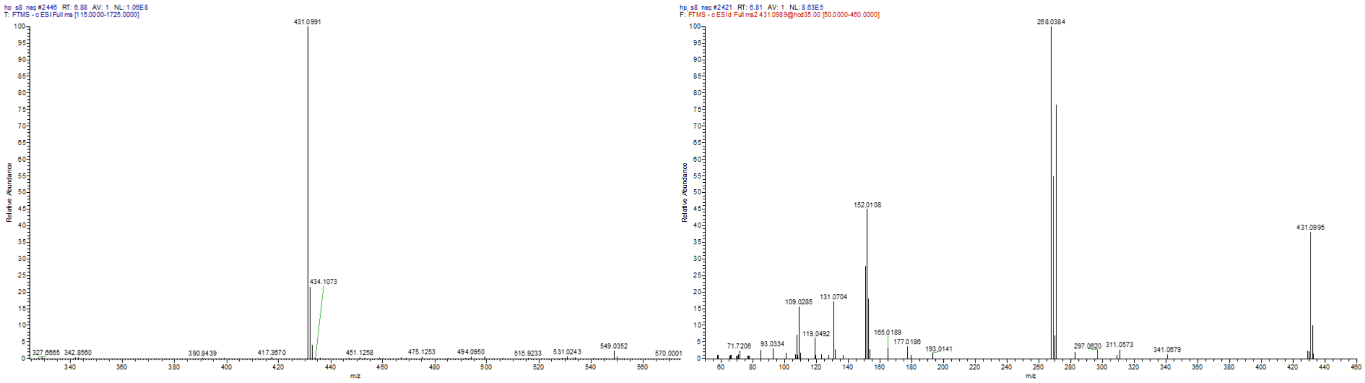

Figure S18: Spectra of **18:** Left: Mass spectrum of **18** (m/z = 431); Right: MS2 spectrum of **18** (at m/z = 431).

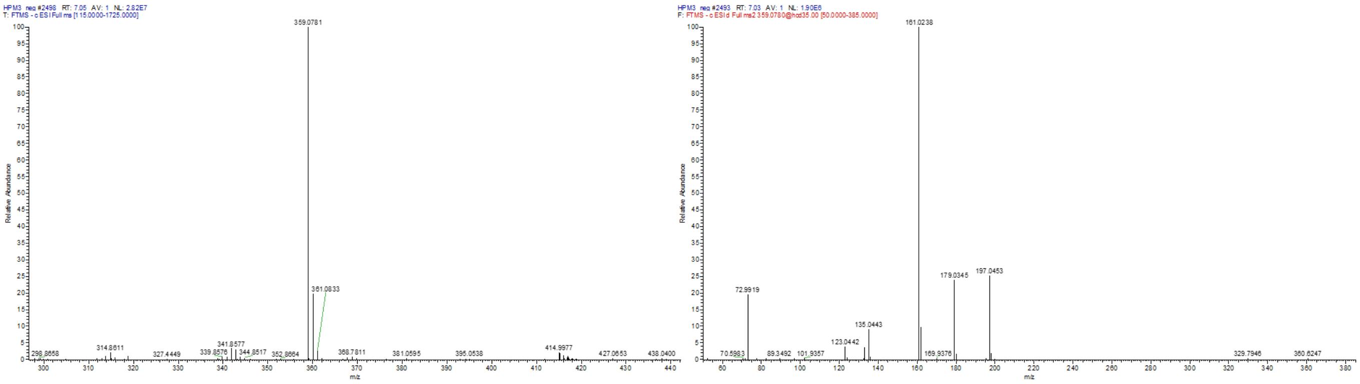

Figure S19: Spectra of **19:** Left: Mass spectrum of **19** (m/z = 359); Right: MS2 spectrum of **19** (at m/z = 359).

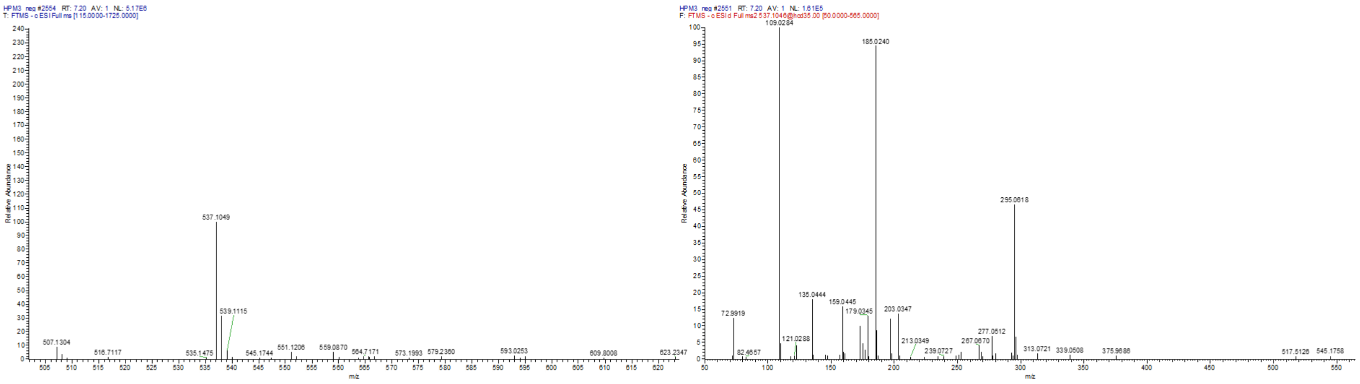

Figure S20: Spectra of **20:** Left: Mass spectrum of **20** (m/z = 537); Right: MS2 spectrum of **20** (at m/z = 537).

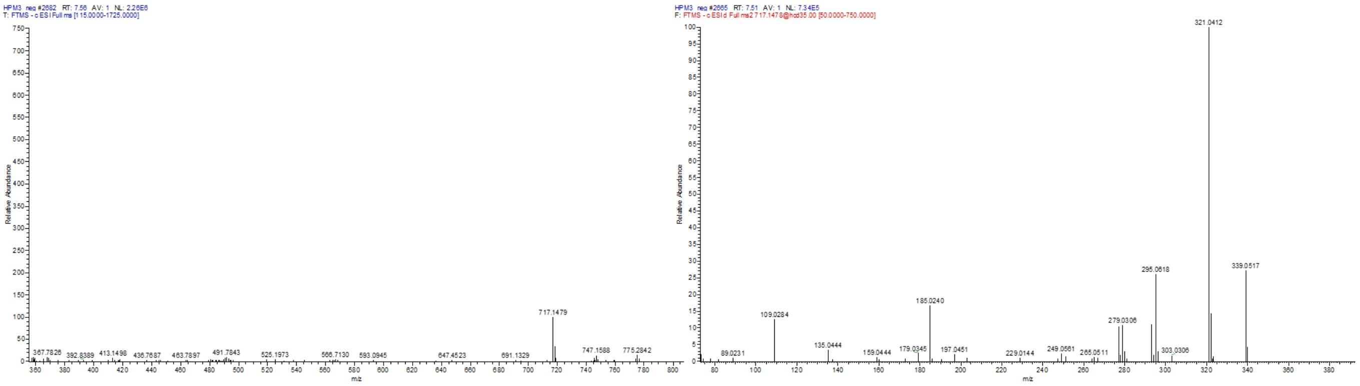

Figure S21: Spectra of **21:** Left: Mass spectrum of **21** (m/z = 717); Right: MS2 spectrum of **21** (at m/z = 717).

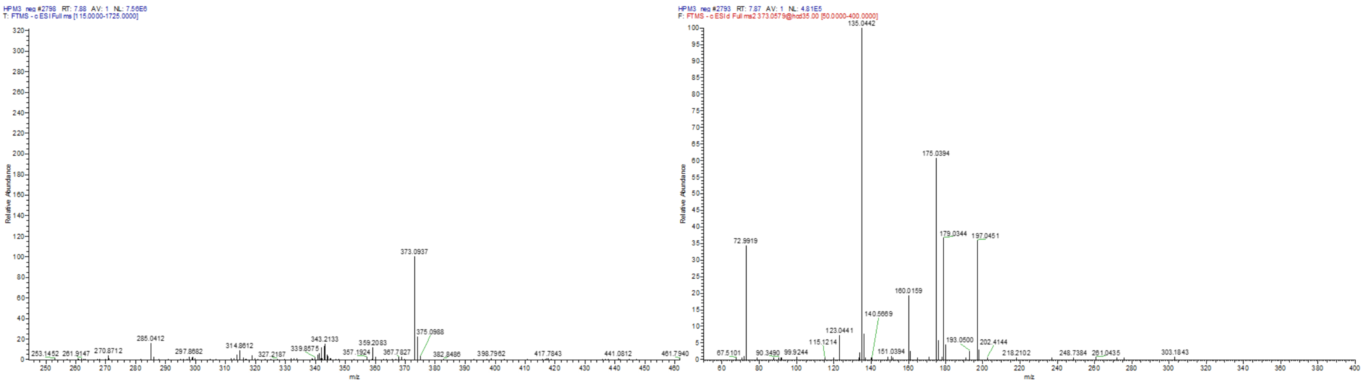

Figure S22: Spectra of **22:** Left: Mass spectrum of **22** (m/z = 373); Right: MS2 spectrum of **22** (at m/z = 373).

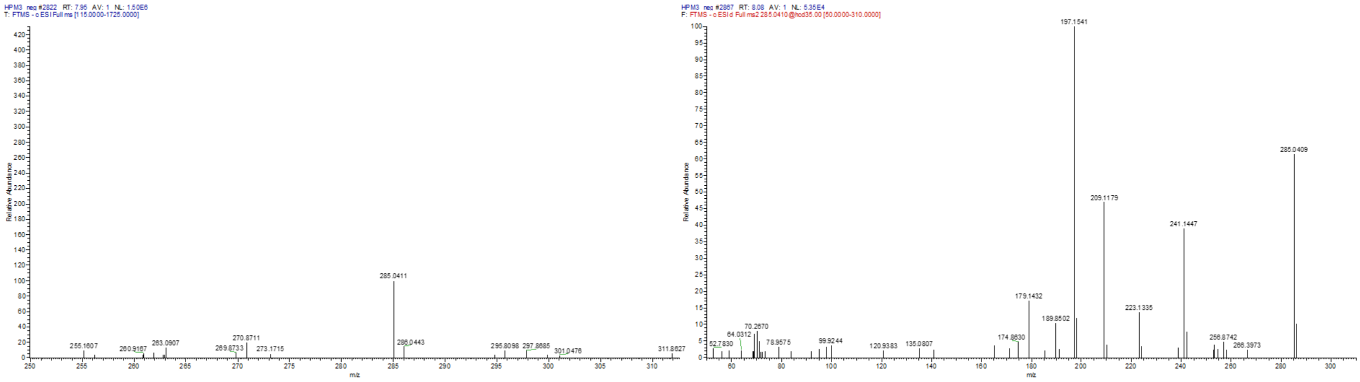

Figure S23: Spectra of **23:** Left: Mass spectrum of **23** (m/z = 285); Right: MS2 spectrum of **23** (at m/z = 285).

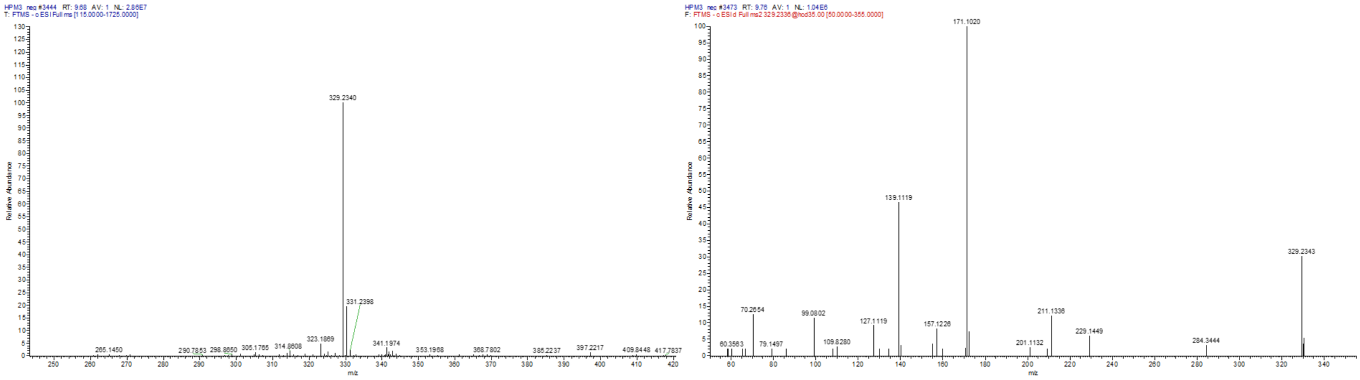

Figure S24: Spectra of **24:** Left: Mass spectrum of **24** (m/z = 329); Right: MS2 spectrum of **24** (at m/z = 329).

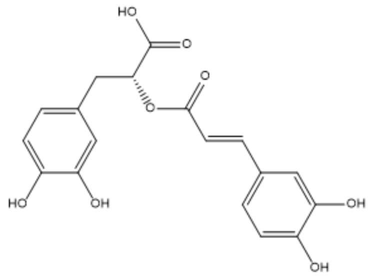

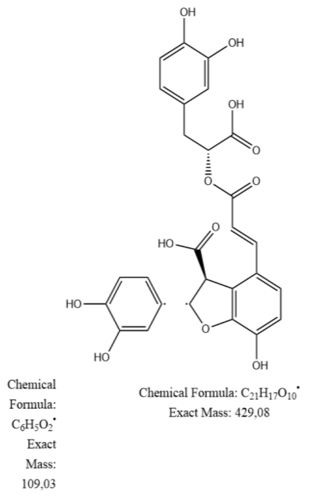

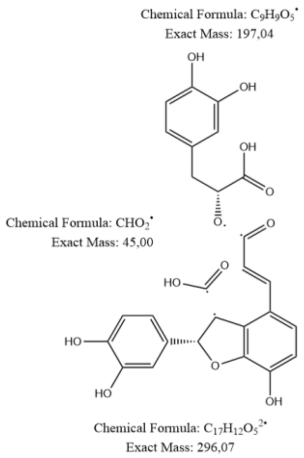

lithospermic acid

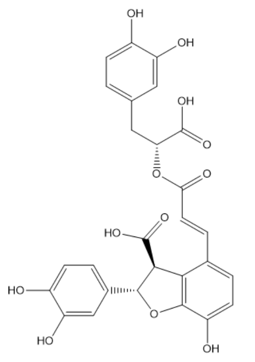

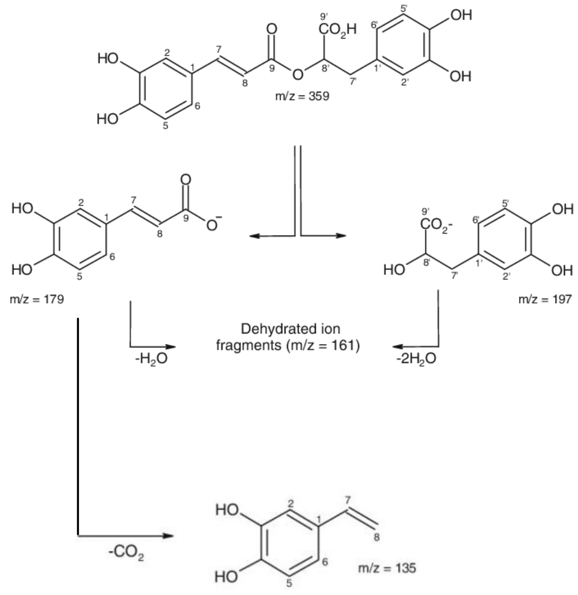

rosmarinic acid

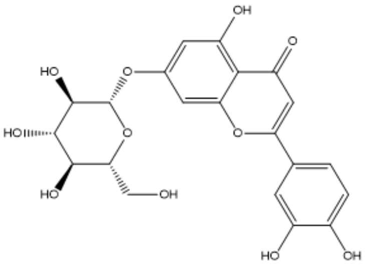

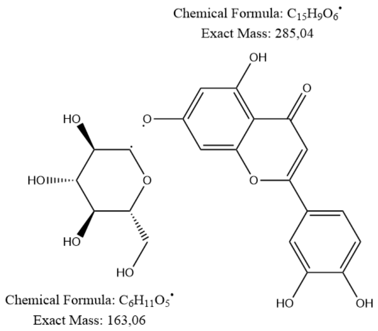

luteolin-7-o-glucosise

Figure S25: The structure and the main fragments of the isolated compounds
